# Impact of Education, Medical Services, and Living Conditions on Health: Evidence from China Health and Nutrition Survey

**DOI:** 10.3390/healthcare9091122

**Published:** 2021-08-30

**Authors:** Xianhua Dai, Wenchao Li

**Affiliations:** 1 School of Public Administration, Central China Normal University, Wuhan 430079, China; liwenchao@mails.ccnu.edu.cn; 2 Center for Labor and Social Security Research, Central China Normal University, Wuhan 430079, China

**Keywords:** education, living conditions, health, medical services, Grossman model

## Abstract

Education, medical services, and living conditions can influence individual health and health literacy. We used the 2015 China Health and Nutrition Survey data to analyze the impact of education, medical services, and living conditions on individual health by extending the Grossman model. As a result, using the instrumental variable (read, write, and draw) two-stage least square method, we found that education, medical services, and living conditions have a positive impact on individual health, both physical health and psychological health. Among them, medical services have the largest influence, followed by living conditions and education. In addition, the results are robust. However, individual characteristics, family income, and working status also affect individual health. Moreover, we observed heterogeneity in age, sex, and residence in the impact of education, medical services, and living conditions on individual health. In particular, the health of the rural elderly and elderly women is more sensitive to education, the medical services of middle-aged women and young men have a greater impact on their health, and the living conditions of the rural elderly and youth have a greater impact on their health. All the findings are helpful for optimizing the path of the Healthy China program.

## 1. Introduction

Health is an important issue in aging societies in many places around the world, and involves many factors, for example, individual characteristics, education, family, medical services, working status, and living conditions. In this study, we explored the influence of education, medical services, and living conditions on individual health, both physical and psychological health.

An area of literature in economics or social science concentrates on the effects of education on health, for example, physical health [1,2,3,4,5] and psychological health [6,7,8,9,10]. These studies either concentrated on the influence mechanism of education on health [11,12,13,14], or how education influences health behavior [15,16,17]. Education affects not only economic income but also health to a certain extent [18]. In those studies, many databases were applied. For example, the China Family Panel Studies [3,12], Chinese Longitudinal Healthy Longevity Survey [17], and Chinese General Social Survey [11]. Differently from these studies, we applied the 2015 China Health and Nutrition Survey data to determine the influence of education on both physical and psychological health. In addition, we used different instrumental variables from other studies and the two-stage least square method to solve the endogenous problem from the model, and verified the robustness of the results by controlling samples and replacing core variables.

Education has a positive effect on health, both physical health and psychological health [9,10,17,19,20]. Specifically, the higher the level of education, the lower the incidence of individual diseases [21], with the disease prevalence rate in those with primary school education or below being 1.64 times that of those with college education or above [22]. For psychological health, education affects individual perception, emotional expression, communication, and interaction abilities [13].

Many other factors also affect physical health, for example, age [2], sex [13,23,24,25], region [11,26], marital status [27,28], and employment [29,30]. Psychological adjustment [17], political education [10], sex and social interaction [7], and peer relationships [31] also affect psychological health. In addition, smoking and drinking [32,33] and family capital affect individual health [34,35]; medical insurance affects both physical [36] and psychological health [37].

Living conditions, for example, running water [38,39,40], toilet access [41,42,43,44], and sanitary conditions [45,46,47,48] also affect individual health. Specifically, having running water and flush toilets not only significantly affects individual physical health [49] but also benefits psychological health [50,51,52]. Sanitary conditions have a positive impact on individual physical and psychological health, but the impacts are slightly different [53,54].

Having medical insurance not only significantly reduces the medical burden of residents [55] and the risk of chronic diseases [56], but also promotes the fair use of medical services [36], improves individual health status [57], and affects individual psychological health [58,59,60]. Whether illness is treated by a doctor has a considerable effect on physical health [61], i.e., positive medical decision making affects the level of physical health [62,63].

Differently from previous studies, we included all these factors in our basic model, and compared, in particular, the influence of education, medical services, and living conditions on individual physical and psychological health. Moreover, using the two-stage least square method, we verified the robustness of the results, and explored the heterogeneity in age, sex, and residence in the effect of education, medical services, and living conditions on individual health.

The remainder of this paper is organized as follows: Section 2 describes the data, variables, and statistics. Section 3 outlines the basic model. Section 4 provides the empirical results and analysis, including the instrumental variable choice, robustness analysis, and heterogeneity analysis. Section 5 draws the study’s conclusions. And Section 6 is for a discussion of the results.

## 2. Data, Variables, and Summary Statistics

### 2.1. Data

We applied the data from the China Health and Nutrition Survey (CHNS), a collaborative project conducted by the Population Center of the University of North Carolina and China Institute of Nutrition and Health [64]. They covered urban and rural areas in 9 provinces, and administered a survey on the dietary structure and nutritional status of residents, also obtaining information on residents’ demographic background, health status, and work situation. In addition, the survey had detailed community data, including information on medical institutions and other social service facilities. The comprehensive and specific characteristics of the survey’s data are suitable for the analysis in this study. It began in 1989 and has been conducted ten times to date. We used the microdata of the 2015 survey, which is the latest and most comprehensive year of the survey to date, selected the variables related to physical and psychological health, education, demographic information, family situation, medical services, habits, working status, and living conditions, and extracted data from different files and integrated them.

#### 2.1.1. Explained Variable

We measured physical health using self-rated health indicators with a correction, including “What do you think of your current health status?”, “Did you have the following symptoms in the past four weeks?”, and “Disease history (including hypertension, diabetes, myocardial infarction, apoplexy, tumor, fracture, and asthma)”. Due to the subjectivity of self-rated health questionnaires, we divided these three indicators in the survey into healthy and unhealthy using a quantile of 60%, then used the median number to correct the self-rated health. The revised self-rated health is physical health, which ranges from 1 to 5 (1, very poor; 2, relatively poor; 3, moderate; 4, relatively good; 5, very good).

We measured psychological health using the test scale in the questionnaire. It contains questions such as “Did you feel like you could not control the important things in your life in the last month?”, “Did you think things were going as you hoped in the last month?”, and “Were you angry about something you could not control in the last month?” We weighted the answers to those three questions, with values ranging from 1 (the worst psychological health) to 5 (the best psychological health).

#### 2.1.2. Explanatory and Control Variables

We measured individual education by years of education. Years of education is a continuous variable and ranges from 0 (never attended school) to 21 (six years of university). For individual characteristics, we included sex, age, residence, and marital status. For sex, 0 indicates women and 1 indicates men. Age is a continuous variable. For residence, 0 indicates rural and 1 indicates urban. For marital status, 0 denotes unmarried and 1 denotes married. For working conditions, we included the job, working hours, and individual income. For living habits, we included smoking and drinking. Working hours and individual income are continuous variables, while job, smoking, and drinking are binary variables (0 for “no” and 1 for “yes”).

We measured medical services using medical insurance (0, without medical insurance; 1, with medical insurance) and medical institution (0 indicating that the individual did not attend a regular hospital for medical treatment, and 1 indicating the individual attended a regular hospital for medical treatment). We measured running water with 0 for “without running water” and 1 for “having running water”; toilet with 0 for “outdoors or no flush” and 1 for “indoors and has a flush”; and sanitary conditions with 0 for “no feces in the house” and 1 for “having feces in the house”. We took the logarithm of the annual income of individuals and families after adding one and dealt with the working hours per week using the Z score. We controlled the sample to “currently not at school” and Han nationality.

### 2.2. Summary Statistics

By deleting invalid values, the final valid samples of physical health and psychological health included 3335 and 3336 samples, respectively. As shown in Table 1, approximately 51% were women, 49% were men, 42% lived rurally, 58% lived in urban areas, and age ranged from 18 to 94 years. The mean values of symptoms and disease history are 0.12 and 0.29, respectively. The average physical health of the sample is 3.83, while the average psychological health is 3.49. The overall level of physical health is higher than the level of psychological health. Few people had a higher education level, and the average education level of the sample is 10.66, which is junior high school. They reported moderate to good physical health and psychological health. The overall living conditions (running water, toilet, and sanitary conditions) of the sample are moderate to good.

As shown in Table A1 in the Appendix A for summary statistics of physical and psychological health (by age), young people reported the highest physical health and psychological health. The physical health of the elderly was the worst and the psychological health of middle-aged people was the worst. The symptom and disease history level of the elderly is evidently higher than that of the other age groups. As shown, the physical function of the elderly decreased with increasing age and middle-aged people reported experiencing more pressure in life. With the popularization of compulsory education, the education level is increasing, and the education level of the young people was found to be significantly higher than that of middle-aged and elderly respondents.

As shown in Table A2 in the Appendix A for summary statistics of physical and psychological health (by age and sex), the physical health and psychological health of young men were the best, while the physical health of elderly women and the psychological health of middle-aged women were the worst.

As shown in Table A3 in the Appendix A for summary statistics of physical and psychological health (by age and residence), the physical health of urban youth was the best, while that of the rural elderly was the worst. The psychological health of the urban elderly was the best, but that of the rural elderly was the worst. Among them, the education levels of young men and women, urban youth, and middle-aged adults were higher. Within the same age groups, the education of men and those in urban areas was higher than that of women and those in rural areas.

## 3. Basic Model

Since individual characteristics, education, family, medical services, working conditions, and living conditions affect individual health, by extending Grossman’s basic health theory, we applied the following econometric model:

(1) Hi=β0+β1Edui+∑k=110βk2Indivki+∑m=13βm3Envirmi+∑n=12βn4Medni+εi

where $H_{i}$ is the health level of individual i, and refers specifically to physical health and psychological health in this paper; $\beta_{0}$ is the intercept term; $Edu_{i}$ is the education level of individual i; $indiv_{ki}$ is the characteristics of individual i (*i* = 1, 2, …, 10); Envirmi is the living conditions of individual i (*m* = 1, 2, 3); $Med_{ni}$ is the medical services (*n* = 1, 2); $\beta_{1}\sim \beta_{16}$ are the corresponding coefficients for those variables; and $\varepsilon_{i}$ is the random error term.

## 4. Empirical Results

### 4.1. Results from the OLS Method

Using the 2015 CHNS data, we applied the ordinary least square (OLS) method to investigate the impact of education, medical services, and living conditions on individual physical health and psychological health after controlling for the individuals currently not at school and of Han nationality. After excluding the influence of collinearity, the obtained results are shown in Table 2.

As shown in Table 2 and Table 3, Model 1 only considers the effect of education on physical and psychological health, where the effect of education on physical health and psychological health is positive. In addition, age has a negative effect on physical health and being male has a negative impact on psychological health. Model 2 further includes family income, where the level of significance remains unchanged (*p* < 0.01). Model 3 further includes medical services, including medical insurance and medical institution. The effect of education on health is still significant. Both medical insurance and medical institution have a significant effect on physical health, but only medical insurance has a significant effect on psychological health. Models 4 and 5 further include individual characteristics and working conditions, respectively. Model 6 further includes habits, including smoking and drinking. Model 7 further includes living conditions (running water, toilet, and sanitary conditions).

As shown in the third and fifth columns of Table A4 in the Appendix A, we include all variables in the model at the same time, and the average effect is 1.1%. Thus, the effect of education on physical and psychological health is positive and remains unchanged at the 1% significance level. For physical and psychological health, after including family income, medical services, working status, and living conditions, the model is better fitted. As shown in Table A5 and Table A6 in the Appendix A, this model considers the net effect of each type of variable on health, where education, individual characteristics, and work status have a greater effect on physical health, whereas education, working status, and living conditions have a greater effect on psychological health. The results are slightly different from those of the hierarchical regression.

### 4.2. Endogeneity Test

As shown in Table 2 and Table 3, education improves the physical and psychological health of individuals, but there may be endogenous problems that lead to the estimated error of $\varepsilon_{i}$. Since we restricted the sample to those currently not at school, a simultaneous causal relationship between education and health does not exist in this model. According to the literature, compulsory education and parents’ education level may be instrumental variables, but they do not work in this model. Therefore, a suitable instrumental variable is chosen from the questionnaire: read, write, and draw (RWD). The regression equation for the first stage of education is as follows:

(2) $$Edu_{i}=\alpha_{0}+\alpha_{1}RWD_{i}+\sum_{k=1}^{10}\alpha_{k2}Indiv_{ki}+\sum_{m=1}^{3}\alpha_{m3}Envir_{mi}+\sum_{n}^{2}\alpha_{n4}Medi_{ni}+\mu_{i}$$

where $RWD_{i}$ is a binary variable, where 0 indicates individuals who do not participate in those activities and 1 indicates those who do; $\alpha_{1}$~$\alpha_{16}$ are the parameters to be estimated. As shown in the second column of Table 4, this instrumental variable is highly relevant to education and RWD does not directly affect physical and psychological health, but affects physical and psychological health indirectly through education.

As shown in the fourth and six columns of Table A4, the average effects of physical and psychological health are 5.8% and 3.7%, respectively (*p* < 0.01 for physical health and *p* < 0.05 for psychological health), but the results from OLS are 1.1% and 1.2%, respectively. Since the Cragg–Donald Wald F statistic should be greater than 10 and the F statistic is 145.88 for physical health and 144.40 for psychological health in this study, we determined that RWD is a suitable instrumental variable. The impacts of sex, medical insurance, medical institution, residence, working hours, running water, and toilets on physical health are all significant, and the impacts of sex, age, medical insurance, and sanitary conditions on psychological health are significant.

### 4.3. Robustness Test

To verify the reliability of the regression results, we conducted a robustness test. At first, we selected another index measuring education using the highest level of education. As shown in the second, third, fourth, and fifth columns of Table 4, regardless of whether the years of education or highest education level is used, it has a positive impact on both physical health and psychological health, and the significance level (1% for physical health and 5% for psychological health) remains unchanged. To avoid the contingency of this method, we reduced the sample size, using 40%, 60%, and 80% random sampling. Limited by space, we only show the random sampling results of 60% in columns 4 and 7. The levels of physical health (0.072, *p* < 0.01) and psychological health (0.043, *p* < 0.05) remain unchanged. Therefore, we determined that the regression results are robust.

### 4.4. Heterogeneity Analysis

This section discusses group differences in the impacts of those factors on health.

#### 4.4.1. Heterogeneity in Age

As shown in Table 5, the effect of education on physical health is significant only for the youth (*p* < 0.1) and elderly (*p* < 0.01), for whom the average effect is 5.5% and 7%, respectively. The effect of education on elderly health is greater than that on the youth. In addition, the effect of medical institution on youth health is significant (*p* < 0.1). At middle age, family income (*p* < 0.05), medical insurance (*p* < 0.01), medical institution (*p* < 0.05), marital status (*p* < 0.05), and individual income (*p* < 0.1) have a significant effect on physical health. Medical insurance has the greatest effect, followed by medical institution and marital status. For the elderly, sex (*p* < 0.05), medical institution (*p* < 0.01), residence (*p* < 0.05), drinking (*p* < 0.05), running water (*p* < 0.01), and toilets (*p* < 0.1) have significant effects on physical health. The effect of medical institution is the largest, followed by running water, residence, drinking, and sex.

As shown in Table 6, education has a significant effect on psychological health (*p* < 0.05) only for the elderly. For the young, sanitary conditions have the greatest impact, followed by family income. At middle age, the effect of medical insurance is the largest, followed by individual income. For the elderly, sex has the greatest effect, followed by medical insurance and family income.

#### 4.4.2. Heterogeneity in Age–Sex and Age–Residence

As shown in Table A7 in the Appendix A for the results of two-stage least squares (by age and sex, for physical health), education has a significant impact on physical health only for middle-aged women, elderly men, and elderly women. Overall, medical services have a greater effect on the physical health of middle-aged men and women, and elderly men and women. Living conditions have a greater effect on the physical health of middle-aged women, and elderly men and women. Medical services have a greater effect on the psychological health of young men and women, the middle-aged, and elderly women.

As shown in Table A8 in the Appendix A for the results of two-stage least squares (by age and sex, for psychological health), education has significant effect on psychological health only for young and elderly women.

As shown in Table A9 in the Appendix A for the results of two-stage least squares (by age and residence, for physical health), education has a significant effect on physical health only for rural youth, rural middle-aged adults, and urban and rural elderly.

As shown in Table A10 in the Appendix A for the results of two-stage least squares (by age and residence, for psychological health), education has a significant effect on psychological health only for the urban youth and urban elderly.

Therefore, the education has the greatest effect on the physical health of rural middle-aged adults and rural elderly, and education has the greatest effect on the psychological health of urban youth and elderly women. Overall, medical services have a greater effect on the physical health of middle-aged and elderly people in urban and rural areas. Living conditions have a greater effect on the physical health of elderly people in urban and rural areas. Medical services have a greater effect on the psychological health of middle-aged and elderly people in urban areas, and living conditions have greater effect on the psychological health of young people in rural areas.

## 5. Conclusions

In this study, we used the data from the 2015 China Health and Nutrition Survey to explore the influence of education, medical services, and living conditions on both individual physical health and psychological health. To avoid the problem of endogeneity, we adopted the two-stage least squares method.

The influence of education on physical and psychological health is significant, positive, and robust, and the average effect of education on physical health is greater than on psychological health. Therefore, education not only significantly improves the level of individual physical health but also increases the positive emotions of individuals, thus having a positive impact on their psychological health.

Many other variables were found to affect physical and psychological health, for example, individual characteristics (including sex, age, and residence), family conditions (family income), medical services (including medical insurance and institutions), working (including working hours and income), and living conditions (including running water, access to a flush toilet, and sanitary conditions).

According to the R^2^ values, the three main factors influencing individual physical and psychological health are education, medical services, and living conditions. Among them, medical services have the largest effect, followed by living conditions and education. In particular, age has a negative impact on physical health; family income, individual income, and working hours have a positive effect on physical health. Being a woman and individual income positively impact psychological health.

We found heterogeneity in age for the impact on physical health and psychological health. Education, for the young and elderly, has a significant impact on their physical health, but only the education level of the elderly has a significant impact on their psychological health. The impact of the education level of the elderly on their physical health is greater than that on their psychological health. Therefore, for older individuals, the impact of education on health should be considered from a longer-term perspective. Similarly, we found heterogeneity in age in the impact of medical services and living conditions on individual physical and psychological health. Medical services have an impact on physical health for all three age groups and on the psychological health of middle-aged and elderly individuals, but living conditions only affect the physical health of the elderly and the psychological health of the young.

For different age–sex and age–residence combinations, education has a positive impact on physical health for middle-aged women, elderly men and women, rural youth, rural middle-aged adults, and urban and rural elderly; the effects for elderly men, rural middle-aged adults, and the rural elderly are much greater. Education has a positive impact on the psychological health only of young and women, urban youth, and the elderly, and the effect on elderly women and urban youth is much greater. Therefore, more attention needs to be paid to elderly men, rural middle-aged adults, rural elderly, elderly women, and urban youth. Similarly, we observed heterogeneity in age–sex and age–residence for the impact of medical services and living conditions on individual physical and psychological health.

## 6. Discussion

There are some limitations to this study. Although an instrumental variable was used, the data in this study were only obtained from a 2015 survey, and some interference problems were unavoidable due to time-varying factors. On this basis, the error terms of the model were assumed to be independently identically distributed. There is a weak imbalance between urban and rural areas in the sample proportion, which may have led to biased estimation. In addition, we concentrated on the impacts of education, medical services, and living conditions on individual physical and psychological health, but those mechanisms need to be further investigated.

The obtained results have policy implications. Investment in improving education, medical services, and living conditions should be increased to improve individual physical and psychological health. The findings provide a reliable theoretical basis for the promotion of the Healthy China strategy. Polices should be formulated in different ways to improve health, including education, medical services, and living conditions. Moreover, health policies should pay attention to the differences between various groups.

## Figures and Tables

**Table 1:** Summary statistics of the variables.

	No.	Min (M)	Max (X)	Average (E)	Standard Error	Standard Deviation	Variance
Self-rated health	3352	1	5	3.75	(0.014)	0.807	0.652
Symptoms	3352	0	6	0.12	(0.009)	0.493	0.243
Disease history	3336	0	4	0.29	(0.010)	0.566	0.321
Physical health	3335	1	5	3.83	(0.012)	0.719	0.517
Psychological health	3336	0.33	5.00	3.49	(0.012)	0.722	0.522
Years of education	3352	0	21	10.66	(0.090)	5.216	27.206
Sex	3352	0	1	0.49	(0.009)	0.500	0.250
Age	3352	18	94	53.06	(0.247)	14.303	204.566
Family income	3352	4.45	14.74	11.08	(0.173)	0.999	0.998
Medical insurance	3352	0	1	0.98	(0.003)	0.147	0.022
Medical institution	3352	0	1	0.04	(0.003)	0.201	0.040
Residence	3352	0	1	0.58	(0.009)	0.493	0.243
Marital status	3352	0	1	0.89	(0.005)	0.312	0.098
Job	3352	0	1	0.55	(0.009)	0.498	0.248
Working hours	3352	−2.04	4.81	0.01	(0.017)	1.000	1.000
Individual income	3352	0.00	14.69	9.90	(0.028)	1.594	2.539
Smoking	3352	0	1	0.27	(0.008)	0.444	0.197
Drinking	3352	0	1	0.29	(0.008)	0.452	0.204
Running water	3352	0	1	0.90	(0.005)	0.304	0.092
Toilet	3352	0	1	0.63	(0.008)	0.483	0.234
Sanitary conditions	3352	0	1	0.89	(0.005)	0.310	0.096

**Table 2:** Results of the impact of education, medical services, and living conditions on physical health.

	Model 1 N = 3335	Model 2 N = 3335	Model 3 N = 3335	Model 4 N = 3335	Model 5 N = 3335	Model 6 N = 3335	Model 7 N = 3335
Intercept Term	3.910 *** (0.065)	3.221 *** (0.155)	3.308 *** (0.172)	3.405 *** (0.178)	3.378 *** (0.79)	3.374 *** (0.179)	3.284 *** (0.183)
Years of education	0.019 *** (0.003)	0.014 *** (0.003)	0.015 *** (0.003)	0.012 *** (0.003)	0.011 *** (0.003)	0.011 *** (0.003)	0.011 *** (0.003)
Sex	0.002 (0.025)	0.021 (0.025)	0.017 (0.025)	0.022 (0.025)	0.012 (0.025)	−0.009 (0.032)	−0.010 (0.032)
Age	−0.005 *** (0.001)	−0.005 *** (0.001)	−0.004 *** (0.001)	−0.005 *** (0.001)	−0.004 *** (0.001)	−0.004 *** (0.001)	−0.004 *** (0.001)
Family income		0.065 *** (0.013)	0.070 *** (0.013)	0.064 *** (0.014)	0.047 *** (0.016)	0.047 *** (0.016)	0.045 *** (0.016)
Medical insurance			−0.164 ** (0.083)	−0.177 ** (0.082)	−0.183 ** (0.082)	−0.184 ** (0.082)	−0.178 ** (0.082)
Medical institution			−0.300 *** (0.061)	−0.325 *** (0.061)	−0.319 *** (0.061)	−0.319 *** (0.061)	−0.315 *** (0.061)
Residence				0.081 ** (0.030)	0.047 (0.031)	0.048 (0.031)	0.030 (0.034)
Marital status				0.007 (0.039)	−0.007 (0.039)	−0.008 (0.039)	−0.007 (0.039)
Job					0.027 (0.032)	0.024 (0.032)	0.026 (0.032)
Working hours					0.026 ** (0.012)	0.025 ** (0.012)	0.025 ** (0.012)
Individual income					0.021 ** (0.010)	0.021 ** (0.010)	0.020 * (0.010)
Smoking						0.007 (0.034)	0.007 (0.034)
Drinking						0.037 (0.033)	0.039 (0.033)
Running water							0.121 *** (0.042)
Toilet							−0.009 (0.032)
Sanitary conditions							0.026 (0.042)
SER	0.704	0.702	0.699	0.698	0.698	0.698	0.697
R^2^	0.042	0.049	0.058	0.059	0.062	0.062	0.065

Note: *, **, and *** indicate significance at 10%, 5%, and 1% levels, respectively; the values in parentheses are the robust standard errors.

**Table 3:** Results of the impact of education, medical services, and living conditions on psychological health.

	Model 1 N = 3336	Model 2 N = 3336	Model 3 N = 3336	Model 4 N = 3336	Model 5 N = 3336	Model 6 N = 3336	Model 7 N = 3336
Intercept Term	3.230 *** (0.067)	2.641 *** (0.157)	2.361 *** (0.176)	3.416 *** (0.182)	2.390 *** (0.183)	2.384 *** (0.184)	2.347 *** (0.188)
Years of education	0.019 *** (0.003)	0.015 *** (0.003)	0.015 *** (0.003)	0.013 *** (0.003)	0.012 *** (0.003)	0.012 *** (0.003)	0.011 *** (0.003)
Sex	−0.051 ** (0.025)	−0.050 ** (0.025)	−0.051 ** (0.025)	−0.049 * (0.025)	−0.060 ** (0.026)	−0.084 ** (0.033)	−0.083 ** (0.033)
Age	0.001 (0.001)	0.02 * (0.001)	0.002 * (0.001)	0.001 (0.001)	0.002 (0.001)	0.002 (0.001)	0.002 (0.001)
Family income		0.056 *** (0.013)	0.054 *** (0.013)	0.049 *** (0.014)	0.027 * (0.016)	0.027 * (0.016)	0.022 (0.016)
Medical insurance			0.308 *** (0.085)	0.302 *** (0.085)	0.291 *** (0.085)	0.290 *** (0.085)	0.287 *** (0.085)
Medical institution			−0.000 (0.063)	−0.004 (0.063)	0.002 (0.063)	0.001 (0.063)	0.001 (0.063)
Residence				0.046 (0.031)	0.030 (0.032)	0.031 (0.032)	−0.002 (0.034)
Marital status				0.040 (0.040)	0.042 (0.040)	0.041 (0.040)	0.048 (0.040)
Job					0.024 (0.032)	0.022 (0.032)	0.023 (0.032)
Working hours					0.009 (0.012)	0.009 (0.012)	0.007 (0.012)
Individual income					0.027 ** (0.010)	0.027 ** (0.010)	0.025 ** (0.010)
Smoking						0.019 (0.035)	0.020 (0.035)
Drinking						0.030 (0.034)	0.031 (0.034)
Running water							0.020 (0.043)
Toilet							0.044 (0.032)
Sanitary conditions							0.113 *** (0.042)
SER	0.716	0.715	0.713	0.713	0.713	0.713	0.712
R^2^	0.017	0.022	0.026	0.027	0.030	0.030	0.033

Note: *, **, and *** indicate significance at 10%, 5%, and 1% levels, respectively; the values in parentheses are robust standard errors.

**Table 4:** Results of the robustness test.

	Physical Health			Psychological Health		
	2SLS1 N = 3335	Robust1 N = 3335	Robust3 N = 1985	2SLS2 N = 3336	Robust2 N = 3336	Robust4 N = 1985
Intercept Term	3.020 *** (0.209)	3.118 *** (0.198)	3.078 *** (0.280)	2.202 *** (0.208)	2.264 *** (0.198)	2.151 *** (0.276)
Years of education/highest education	0.058 *** (0.016)	0.179 *** (0.049)	0.072 *** (0.020)	0.037 ** (0.016)	0.116 ** (0.049)	0.043 ** (0.020)
Sex	−0.084 ** (0.041)	−0.073 * (0.039)	−0.112 ** (0.055)	−0.125 *** (0.041)	−0.118 *** (0.040)	−0.149 *** (0.054)
Age	0.001 (0.002)	0.001 (0.002)	0.003 (0.003)	0.005 ** (0.002)	0.004 ** (0.002)	0.005 * (0.003)
Family income	0.028 (0.017)	0.031 * (0.017)	0.025 (0.022)	0.012 (0.017)	0.014 (0.017)	0.008 (0.021)
Medical insurance	−0.167 * (0.085)	−0.157 * (0.085)	−0.200 * (0.113)	0.290 *** (0.086)	0.297 *** (0.086)	0.433 *** (0.111)
Medical institution	−0.325 *** (0.063)	−0.323 *** (0.063)	−0.367 *** (0.081)	−0.006 (0.064)	−0.005 (0.064)	0.045 (0.080)
Residence	−0.146 ** (0.068)	−0.148 ** (0.068)	−0.177 ** (0.086)	−0.102 (0.068)	−0.103 (0.069)	−0.088 (0.085)
Marital status	−0.007 (0.041)	−0.006 (0.040)	−0.054 (0.054)	0.048 (0.041)	0.049 (0.041)	0.001 (0.053)
Job	−0.009 (0.035)	−0.020 (0.036)	−0.030 (0.047)	0.004 (0.035)	−0.003 (0.036)	−0.008 (0.046)
Working hours	0.026 ** (0.013)	0.024 * (0.012)	0.018 (0.017)	0.007 (0.013)	0.006 (0.013)	0.008 (0.016)
Individual income	0.005 (0.012)	0.006 (0.011)	−0.007 (0.016)	0.016 (0.012)	0.016 (0.011)	0.012 (0.015)
Smoking	0.051 (0.038)	0.047 (0.038)	0.037 (0.051)	0.045 (0.038)	0.042 (0.038)	0.030 (0.050)
Drinking	0.040 (0.034)	0.042 (0.034)	0.055 (0.045)	0.032 (0.034)	0.033 (0.034)	0.057 (0.044)
Running water	0.132 *** (0.044)	0.128 *** (0.044)	0.135 ** (0.061)	0.026 (0.044)	0.024 (0.044)	0.051 (0.059)
Toilet	−0.070 * (0.038)	−0.065 * (0.038)	−0.070 (0.051)	0.008 (0.039)	0.011 (0.038)	−0.020 (0.050)
Sanitary conditions	−0.006 (0.044)	−0.003 (0.044)	−0.037 (0.062)	0.095 ** (0.044)	0.097 ** (0.044)	0.060 (0.061)
SER	0.720	0.717	0.732	0.719	0.719	0.719
R^2^	0.003	0.011	−0.046	0.013	0.012	0.012
F	145.88	155.86	89.62	144.40	154.65	88.44

Note: *, **, and *** indicate significance at the 10%, 5%, and 1% levels, respectively; the values in brackets are robust standard errors.

**Table 5:** Results of two-stage least squares (by age, for physical health).

	Full Sample N = 3335	Youth N = 1022	Middle-Aged N = 1119	Elderly N = 1194
Intercept Term	3.105 *** (0.177)	3.629 *** (0.329)	2.582 *** (0.302)	3.054 *** (0.292)
Years of education/RWD	0.057 *** (0.015)	0.055 * (0.031)	0.053 (0.036)	0.070 *** (0.022)
Sex	−0.080 ** (0.037)	−0.035 (0.061)	−0.038 (0.085)	−0.174 ** (0.068)
Family income	0.026 (0.018)	0.002 (0.041)	0.066 ** (0.029)	0.020 (0.027)
Medical insurance	−0.165 * (0.085)	0.024 (0.142)	−0.421 *** (0.148)	−0.124 (0.158)
Medical institution	−0.320 *** (0.063)	−0.472 * (0.256)	−0.263 ** (0.111)	−0.342 *** (0.082)
Residence	−0.142 ** (0.062)	−0.142 (0.120)	−0.089 (0.140)	−0.216 ** (0.103)
Marital status	−0.005 (0.041)	−0.072 (0.079)	0.253 ** (0.107)	−0.060 (0.070)
Job	−0.028 (0.050)	0.065 (0.101)	−0.067 (0.070)	0.071 (0.060)
Working hours	0.025 ** (0.013)	0.021 (0.024)	0.034 (0.022)	0.017 (0.020)
Individual income	0.005 (0.011)	−0.036 (0.024)	0.030 * (0.018)	−0.005 (0.021)
Smoking	0.051 (0.038)	0.093 (0.081)	0.018 (0.073)	0.061 (0.057)
Drinking	0.040 (0.034)	0.028 (0.064)	−0.049 (0.061)	0.125 ** (0.056)
Running water	0.132 *** (0.044)	−0.009 (0.080)	0.097 (0.073)	0.304 *** (0.080)
Toilet	−0.069 * (0.038)	−0.036 (0.082)	−0.052 (0.065)	−0.111 * (0.060)
Sanitary conditions	−0.007 (0.045)	0.048 (0.084)	−0.103 (0.077)	0.061 (0.074)
SER	0.719	0.704	0.733	0.721
R^2^	0.004	−0.031	0.023	−0.018
F	145.337	40.663	33.531	63.905

Note: *, **, and *** indicate significance at the 10%, 5%, and 1% levels, respectively; the values in parentheses are robust standard errors.

**Table 6:** Results of two- stage least squares (by age, for psychological health).

	Full Sample N = 3336	Youth N = 1024	Middle Age N = 1120	Old Age N = 1192
Intercept Term	2.537 *** (0.178)	3.098 *** (0.331)	2.422 *** (0.305)	2.240 *** (0.290)
Years of education/RWD	0.035 ** (0.015)	0.048 (0.032)	0.002 (0.036)	0.047 ** (0.022)
Sex	−0.108 *** (0.037)	−0.013 (0.061)	−0.084 (0.086)	−0.228 *** (0.067)
Family income	0.005 (0.018)	−0.088 ** (0.041)	0.022 (0.029)	0.063 ** (0.027)
Medical insurance	0.296 *** (0.086)	0.229 (0.145)	0.304 ** (0.150)	0.342 ** (0.156)
Medical institution	0.014 (0.063)	0.434 * (0.255)	−0.049 (0.113)	−0.050 (0.081)
Residence	−0.086 (0.063)	−0.212 * (0.122)	0.019 (0.139)	−0.048 (0.102)
Marital status	0.057 (0.041)	0.086 (0.078)	−0.003 (0.108)	0.038 (0.069)
Job	−0.069 (0.049)	0.155 (0.101)	0.023 (0.069)	0.049 (0.059)
Working hours	0.007 (0.013)	−0.022 (0.024)	0.017 (0.023)	0.020 (0.020)
Individual income	0.018 (0.011)	0.026 (0.024)	0.039 ** (0.018)	−0.020 (0.021)
Smoking	0.046 (0.038)	−0.037 (0.081)	0.038 (0.073)	0.104* (0.057)
Drinking	0.032 (0.034)	0.067 (0.064)	0.010 (0.061)	0.021 (0.056)
Running water	0.026 (0.044)	0.047 (0.080)	0.009 (0.073)	0.042 (0.079)
Toilet	0.012 (0.038)	−0.001 (0.084)	0.007 (0.065)	0.001 (0.059)
Sanitary conditions	0.092 ** (0.044)	0.186 ** (0.083)	0.069 (0.077)	0.064 (0.073)
SER	0.719	0.701	0.740	0.713
R^2^	0.012	0.022	0.024	0.013
F	144.220	38.592	34.884	63.242

Note: *, **, and *** indicate significance at 10%, 5%, and 1%, levels, respectively; the values in parentheses are robust standard errors.

## Data Availability

Data used in this paper can be found from the China Health and Nutrition Survey, https://www.cpc.unc.edu/projects/china.

## Appendix A

**Table A1:** Summary statistics of physical and psychological health (by age).

	Youth	Middle Age	Old Age
Self-rated health	3.92 (0.024)	3.75 (0.024)	3.60 (0.024)
Symptoms	0.07 (0.011)	0.11 (0.013)	0.18 (0.018)
Disease history	0.06 (0.007)	0.22 (0.015)	0.54 (0.021)
Physical health	3.97 (0.022)	3.82 (0.022)	3.72 (0.021)
Psychological health	3.534 (0.022)	3.43 (0.022)	3.50 (0.021)
Years of education	13.22 (0.153)	10.41 (0.134)	8.69 (0.150)
Sex	0.46 (0.016)	0.50 (0.015)	0.51 (0.014)
Age	36.00 (0.215)	52.78 (0.119)	67.91 (0.195)
Family income	11.32 (0.290)	11.08 (0.029)	10.87 (0.030)
Medical insurance	0.97 (0.005)	0.98 (0.004)	0.98 (0.004)
Medical institution	0.01 (0.003)	0.04 (0.006)	0.07 (0.007)
Residence	0.57 (0.015)	0.54 (0.015)	0.63 (0.014)
Marital status	0.86 (0.011)	0.95 (0.006)	0.86 (0.010)
Job	0.89 (0.010)	0.64 (0.014)	0.17 (0.011)
Working hours	0.04 (0.030)	−0.04 (0.030)	0.01 (0.030)
Individual income	10.22 (0.043)	9.87 (0.051)	9.66 (0.047)
Smoking	0.23 (0.013)	0.29 (0.014)	0.28 (0.013)
Drinking	0.26 (0.014)	0.32 (0.014)	0.27 (0.013)
Running water	0.91 (0.009)	0.88 (0.010)	0.90 (0.009)
Toilet	0.67 (0.015)	0.59 (0.015)	0.64 (0.014)
Sanitary conditions	0.91 (0.009)	0.89 (0.009)	0.88 (0.009)

**Table A2:** Summary statistics of physical and psychological health (by age and sex).

	Young Men	Young Women	Middle-Aged Men	Middle-Aged Women	Elderly Men	Elderly Women
Self-rated health	3.95 (0.034)	3.89 (0.033)	3.76 (0.035)	3.75 (0.033)	3.63 (0.034)	3.57 (0.034)
Symptoms	0.07 (0.013)	0.08 (0.017)	0.11 (0.020)	0.11 (0.018)	0.13 (0.020)	0.23 (0.030)
Disease history	0.07 (0.011)	0.05 (0.010)	0.24 (0.021)	0.21 (0.020)	0.55 (0.029)	0.54 (0.029)
Physical health	3.99 (0.032)	3.95 (0.029)	3.83 (0.031)	3.80 (0.031)	3.75 (0.029)	3.69 (0.029)
Psychological health	3.55 (0.031)	3.52 (0.3307)	3.42 (0.031)	3.44 (0.032)	3.47 (0.029)	3.53 (0.030)
Years of education	13.41 (0.218)	13.05 (0.213)	11.27 (0.175)	9.57 (0.196)	9.71 (0.198)	7.62 (0.220)
Family income	11.35 (0.042)	11.29 (0.040)	11.12 (0.041)	11.04 (0.042)	10.91 (0.040)	10.84 (0.044)
Medical insurance	0.99 (0.006)	0.97 (0.008)	0.98 (0.006)	0.98 (0.006)	0.98 (0.006)	0.98 (0.005)
Medical institution	0.01 (0.004)	0.01 (0.004)	0.04 (0.008)	0.04 (0.009)	0.06 (0009)	0.09 (0.012)
Residence	0.57 (0.023)	0.56 (0.021)	0.55 (0.021)	0.54 (0.021)	0.62 (0.020)	0.64 (0.020)
Marital status	0.86 (0.016)	0.86 (0.015)	0.96 (0.008)	0.94 (0.010)	0.91 (0.011)	0.81 (0.016)
Job	0.92 (0.012)	0.86 (0.015)	0.80 (0.017)	0.47 (0.021)	0.23 (0.017)	0.12 (0.013)
Working hours	0.03 (0.044)	0.05 (0.040)	−0.03 (0.043)	−0.05 (0.041)	0.03 (0.043)	−0.02 (0.042)
Individual income	10.38 (0.063)	10.09 (0.057)	10.10 (0.070)	9.63 (0.074)	9.84 (0.057)	9.48 (0.074)
Smoking	0.50 (0.023)	0.01 (0.004)	0.56 (0.021)	0.03 (0.007)	0.50 (0.020)	0.05 (0.009)
Drinking	0.52 (0.023)	0.04 (0.009)	0.60 (0.021)	0.05 (0.009)	0.48 (0.020)	0.05 (0.009)
Running water	0.90 (0.014)	0.91 (0.012)	0.90 (0.013)	0.86 (0.014)	0.90 (0.012)	0.91 (0.012)
Toilet	0.67 (0.022)	0.66 (0.020)	0.61 (0.021)	0.56 (0.021)	0.64 (0.019)	0.64 (0.020)
Sanitary conditions	0.90 (0.014)	0.91 (0.012)	0.89 (0.013)	0.88 (0.014)	0.88 (0.013)	0.88 (0.013)

**Table A3:** Summary statistics of physical and psychological health (by age and residence).

	Urban Youth	Rural Youth	Urban Middle Age	Rural Middle Age	Urban Elderly	Rural Elderly
Self-rated health	3.97 (0.032)	3.84 (0.036)	3.85 (0.032)	3.64 (0.035)	3.66 (0.030)	3.49 (0.039)
Symptoms	0.09 (0.016)	0.05 (0.013)	0.09 (0.017)	0.13 (0.021)	0.17 (0.023)	0.20 (0.029)
Disease history	0.05 (0.010)	0.06 (0.012)	0.22 (0.020)	0.22 (0.021)	0.61 (0.027)	0.42 (0.031)
Physical health	4.03 (0.028)	3.89 (0.033)	3.90 (0.029)	3.71 (0.033)	3.77 (0.026)	3.64 (0.033)
Psychological health	3.57 (0.030)	3.48 (0.032)	3.48 (0.031)	3.37 (0.032)	3.59 (0.026)	3.35 (0.034)
Years of education	15.65 (0.164)	10.05 (0.195)	12.55 (0.155)	7.86 (0.168)	10.72 (0.173)	5.24 (0.190)
Sex	0.47 (0.021)	0.46 (0.024)	0.50 (0.020)	0.49 (0.022)	0.51 (0.018)	0.52 (0.024)
Family income	11.58 (0.035)	10.97 (0.044)	11.31 (0.030)	10.80 (0.051)	11.22 (0.026)	10.28 (0.056)
Medical insurance	0.98 (0.006)	0.97 (0.009)	0.98 (0.006)	0.97 (0.007)	0.98 (0.005)	0.98 (0.007)
Medical institution	0.01 (0.005)	0.00 (0.002)	0.05 (0.009)	0.03 (0.007)	0.10 (0.011)	0.03 (0.008)
Marital status	00.83 (0.015)	0.89 (0.015)	0.94 (0.010)	0.96 (0.008)	0.87 (0.012)	0.84 (0.017)
Job	0.98 (0.006)	0.78 (0.020)	0.66 (0.019)	0.61 (0.022)	0.08 (0.010)	0.33 (0.022)
Working hours	0.089 (0.040)	−0.03 (0.045)	0.03 (0.040)	−0.14 (0.045)	0.08 (0.037)	−0.10 (0.050)
Individual income	10.63 (0.042)	9.69 (0.075)	10.45 (0.032)	9.17 (0.097)	10.34 (0.027)	8.52 (0.095)
Smoking	0.22 (0.017)	0.26 (0.021)	0.28 (0.018)	0.31 (0.021)	0.24 (0.015)	0.35 (0.023)
Drinking	0.25 (0.018)	0.28 (0.021)	0.33 (0.019)	0.31 (0.021)	0.24 (0.016)	0.32 (0.022)
Running water	0.97 (0.007)	0.82 (0.018)	0.97 (0.007)	0.78 (0.018)	0.97 (0.006)	0.79 (0.019)
Toilet	0.87 (0.014)	0.40 (0.023)	0.84 (0.015)	0.28 (0.020)	0.85 (0.013)	0.28 (0.021)
Sanitary conditions	0.98 (0.006)	0.082 (0.018)	0.94 (0.009)	0.82 (0.017)	0.97 (0.006)	0.74 (0.021)

**Table A4:** Results obtained from ordinary least squares and two-stage least squares.

	Education N = 3352	Physical N = 3335	2SLS1 N = 3335	Psychological N = 3336	2SLS2 N = 3336
Intercept Term	6.140 *** (0.980)	3.284 *** (0.183)	3.020 *** (0.209)	2.347 *** (0.188)	2.202 *** (0.208)
Years of education/RWD	1.939 *** (0.160)	0.011 *** (0.003)	0.058 *** (0.016)	0.011 *** (0.003)	0.037 ** (0.016)
Sex	1.513 *** (0.170)	−0.010 (0.032)	−0.084 ** (0.041)	−0.083 ** (0.033)	−0.125 *** (0.041)
Age	−0.119 *** (0.006)	−0.004 *** (0.001)	0.001 (0.002)	0.002 (0.001)	0.005 ** (0.002)
Family income	0.323 *** (0.084)	0.045 *** (0.016)	0.028 (0.017)	0.022 (0.016)	0.012 (0.017)
Medical insurance	−0.131 (0.443)	−0.178 ** (0.082)	−0.167 * (0.085)	0.287 *** (0.085)	0.290 *** (0.086)
Medical institution	−0.131 (0.443)	−0.315 *** (0.061)	−0.325 *** (0.063)	0.001 (0.063)	−0.006 (0.064)
Residence	3.325 *** (0.172)	0.030 (0.034)	−0.146 ** (0.068)	−0.002 (0.034)	−0.102 (0.068)
Marital status	0.041 (0.211)	−0.007 (0.039)	−0.007 (0.041)	0.048 (0.040)	0.048 (0.041)
Job	0.743 *** (0.170)	0.026 (0.032)	−0.009 (0.035)	0.023 (0.032)	0.004 (0.035)
Working hours	−0.027 (0.065)	0.025 ** (0.012)	0.026 ** (0.013)	0.007 (0.012)	0.007 (0.013)
Individual income	0.290 *** (0.054)	0.020 * (0.010)	0.005 (0.012)	0.025 ** (0.010)	0.016 (0.012)
Smoking	−0.847 *** (0.182)	0.007 (0.034)	0.051 (0.038)	0.020 (0.035)	0.045 (0.038)
Drinking	−0.067 (0.177)	0.039 (0.033)	0.040 (0.034)	0.031 (0.034)	0.032 (0.034)
Running water	−0.193 (0.228)	0.121 *** (0.042)	0.132 *** (0.044)	0.020 (0.043)	0.026 (0.044)
Toilet	1.130 *** (0.169)	−0.009 (0.032)	−0.070 * (0.038)	0.044 (0.032)	0.008 (0.039)
Sanitary conditions	0.646 *** (0.223)	0.026 (0.042)	−0.006 (0.044)	0.113 *** (0.042)	0.095 ** (0.044)
SER	3.745	0.697	0.720	0.712	0.719
R^2^	0.487	0.065	0.003	0.033	0.013
F			145.88		144.40

Note: *, **, and *** indicate significance at the 10%, 5%, and 1% levels, respectively; the values in parentheses are robust standard errors.

**Table A5:** Results for physical health.

	Model 1 N = 3335	Model 2 N = 3335	Model 3 N = 3335	Model 4 N = 3335	Model 5 N = 3335	Model 6 N = 3335	Model 7 N = 3335
Intercept Term	3.567 *** (0.028)	4.155 *** (0.060)	2.621 *** (0.137)	4.005 *** (0.083)	3.222 *** (0.077)	3.820 *** (0.015)	3.571 *** (0.048)
Years of education	0.025 *** (0.002)						
Sex		0.046 * (0.025)					
Age		−0.008 *** (0.001)					
Residence		0.156 *** (0.025)					
Marital status		0.010 (0.039)					
Family income			0.109 *** (0.012)				
Medical insurance				−0.164 * (0.084)			
Medical institution				−0.349 *** (0.062)			
Job					0.149 *** (0.025)		
Working hours					0.031 ** (0.012)		
Individual income					0.053 *** (0.008)		
Smoking						−0.028 (0.032)	
Drinking						0.060 * (0.032)	
Running water							0.148 *** (0.043)
Toilet							0.096 *** (0.028)
Sanitary conditions							0.073 * (0.042)
SER	0.708	0.706	0.711	0.715	0.707	0.719	0.715
R^2^	0.032	0.038	0.023	0.011	0.033	0.001	0.013

Note: *, **, and *** indicate significance at the 10%, 5%, and 1% levels, respectively; the values in parentheses are robust standard errors; all samples were controlled to be not in school and Han nationality. The R^2^ values of Models 1, 2, and 5 are better; the R^2^ values of Models 3, 4, and 7 are worse; and the R^2^ of Model 6 is the worst. Individual characteristics (including sex, age, and residence), working conditions (including job, working hours, and individual income), and education have a stronger impact on physical health, followed by family income, living conditions (including running water, toilet access, and sanitary conditions), and medical services (including medical insurance and medical institutions), where living habits (drinking) have the least impact.

**Table A6:** Results for psychological health.

	Model 1 N = 3336	Model 2 N = 3336	Model 3 N = 3336	Model 4 N = 3336	Model 5 N = 3336	Model 6 N = 3336	Model 7 N = 3336
Intercept Term	3.302 *** (0.028)	3.437 *** (0.061)	2.586 *** (0.139)	3.168 *** (0.084)	2.933 *** (0.078)	3.490 *** (0.015)	3.245 *** (0.048)
Years of education	0.017 *** (0.002)						
Sex		−0.027 (0.025)					
Age		−0.002 * (0.001)					
Residence		0.153 *** (0.025)					
Marital status		0.068 * (0.040)					
Family income			0.081 *** (0.012)				
Medical insurance				0.325 *** (0.085)			
Medical institution				0.030 (0.062)			
Job					0.018 (0.026)		
Working hours					0.011 (0.012)		
Individual income					0.055 *** (0.008)		
Smoking						−0.035 (0.032)	
Drinking						0.021 (0.032)	
Running water							0.035 (0.043)
Toilet							0.128 *** (0.028)
Sanitary conditions							0.145 *** (0.042)
SER	0.717	0.718	0.718	0.721	0.717	0.722	0.717
R^2^	0.016	0.012	0.013	0.004	0.016	0.001	0.016

Note: *, **, and *** indicate significance at the 10%, 5%, and 1% levels, respectively; the values in parentheses are robust standard errors; all samples were controlled to be not in school and Han nationality. The R^2^ values of Models 1, 5, and 7 are better; the R^2^ values of Models 2, 3, and 4 are worse; and the R^2^ of Model 6 is the worst. Education, working conditions (individual income), and living conditions (including toilet and sanitary conditions) have a stronger impact on psychological health, followed by family income, individual characteristics (including sex, age, and residence), and medical services (including medical insurance and medical institution), where the impact of living habits is the lowest.

**Table A7:** Results of two-stage least squares (by age and sex, for physical health).

	Full Sample N = 3335	Young Men N = 473	Young Women N = 549	Middle-Aged Men N = 555	Middle-Aged Women N = 564	Elderly Men N = 613	Elderly Women N = 581
Intercept Term	3.095 *** (0.176)	3.244 *** (0.732)	4.052 *** (0.422)	2.208 *** (0.580)	3.281 *** (0.454)	3.025 *** (0.433)	3.019 *** (0.408)
Years of education	0.056 *** (0.015)	0.139 (0.101)	0.033 (0.031)	0.011 (0.070)	0.077 * (0.042)	0.082 ** (0.035)	0.067 ** (0.028)
Family income	0.028 (0.018)	−0.095 (0.140)	0.006 (0.044)	0.093 ** (0.040)	0.012 (0.046)	0.046 (0.046)	−0.003 (0.036)
Medical insurance	−0.165 * (0.085)	0.202 (0.321)	0.018 (0.167)	−0.345 * (0.202)	−0.513 ** (0.225)	−0.145 (0.218)	−0.060 (0.241)
Medical institution	−0.318 *** (0.063)	−0.589 (0.459)	−0.507 (0.350)	−0.394 ** (0.167)	−0.196 (0.162)	−0.430 *** (0.131)	−0.282 *** (0.107)
Residence	−0.139 ** (0.062)	−0.401 (0.343)	−0.077 (0.131)	−0.010 (0.195)	−0.150 (0.213)	−0.148 (0.136)	−0.318 (0.157)
Marital status	−0.010 (0.040)	0.019 (0.149)	−0.122 (0.104)	0.340 ** (0.165)	0.245 (0.150)	−0.143 (0.109)	0.004 (0.097)
Job	−0.028 (0.050)	−0.175 (0.244)	0.161 (0.113)	0.003 (0.128)	−0.099 (0.089)	0.118 (0.079)	−0.014 (0.100)
Working hours	0.025 ** (0.013)	0.028 (0.046)	0.035 (0.031)	0.029 (0.031)	0.043 (0.034)	0.032 (0.029)	−0.002 (0.029)
Individual income	0.003 (0.011)	0.023 (0.044)	−0.067 * (0.035)	0.061 ** (0.028)	0.003 (0.024)	−0.062 * (0.035)	0.025 (0.027)
Smoking	0.017 (0.033)	0.232 (0.171)	0.052 (0.348)	−0.025 (0.103)	0.033 (0.192)	0.088 (0.066)	−0.029 (0.147)
Drinking	0.010 (0.032)	−0.028 (0.096)	0.070 (0.149)	−0.026 (0.064)	−0.218 (0.160)	0.068 (0.064)	0.412 *** (0.147)
Running water	0.131 *** (0.044)	−0.034 (0.145)	−0.001 (0.112)	−0.011 (0.106)	0.182 * (0.105)	0.316 *** (0.112)	0.284 ** (0.118)
Toilet	−0.068 * (0.038)	−0.223 (0.216)	0.029 (0.099)	0.015 (0.100)	−0.128 (0.093)	−0.160 * (0.086)	−0.072 (0.084)
Sanitary conditions	−0.007 (0.044)	−0.034 (0.167)	0.050 (0.112)	−0.105 (0.129)	−0.069 (0.105)	0.150 (0.109)	−0.002 (0.105)
SER	0.719	0.827	0.686	0.705	0.770	0.744	0.711
R^2^	0.006	−0.373	0.015	0.101	−0.060	−0.062	0.007

Note: *, **, and *** indicate significance at the 10%, 5%, and 1% levels, respectively; the values in parentheses are robust standard errors.

**Table A8:** Results of two-stage least squares (by age and sex, for psychological health).

	Full Sample N = 3336	Young Men N = 474	Young Women N = 550	Middle-Aged Men N = 557	Middle-Aged Women N = 563	Elderly Men N = 612	Elderly Women N = 580
Intercept Term	2.526 *** (0.177)	2.778 *** (0.691)	3.520 *** (0.446)	2.300 *** (0.587)	2.257 *** (0.437)	2.371 *** (0.408)	1.816 *** (0.442)
Years of education	0.034 ** (0.015)	0.041 (0.085)	0.060 * (0.033)	0.025 (0.069)	−0.010 (0.041)	0.012 (0.033)	0.084 *** (0.030)
Family income	0.008 (0.018)	−0.078 (0.117)	−0.098 ** (0.047)	0.045 (0.043)	0.011 (0.045)	0.056 (0.044)	0.092 ** (0.039)
Medical insurance	0.296 *** (0.086)	0.015 (0.315)	0.389 ** (0.177)	0.141 (0.213)	0.494 ** (0.218)	0.071 (0.204)	0.621 ** (0.261)
Medical institution	0.017 (0.063)	0.846 ** (0.374)	−0.019 (0.370)	−0.067 (0.178)	−0.043 (0.157)	−0.083 (0.122)	−0.008 (0.117)
Residence	−0.082 (0.062)	−0.209 (0.291)	−0.220 (0.139)	−0.056 (0.194)	0.088 (0.207)	0.085 (0.127)	−0.260 (0.173)
Marital status	0.051 (0.041)	0.115 (0.115)	0.030 (0.111)	−0.034 (0.174)	0.002 (0.146)	0.168 * (0.102)	−0.113 (0.106)
Job	−0.070 (0.049)	0.345 * (0.195)	0.035 (0.119)	0.081 (0.129)	−0.011 (0.087)	−0.012 (0.074)	0.129 (0.107)
Working hours	0.006 (0.013)	−0.062 (0.039)	0.018 (0.033)	0.031 (0.032)	−0.002 (0.032)	0.042 (0.027)	−0.004 (0.032)
Individual income	0.016 (0.011)	0.049 (0.035)	−0.014 (0.038)	0.013 (0.029)	0.055 ** (0.023)	−0.003 (0.032)	−0.045 (0.029)
Smoking	−0.001 (0.033)	−0.038 (0.144)	0.061 (0.368)	0.100 (0.108)	−0.216 (0.185)	0.091 (0.061)	0.157 (0.163)
Drinking	−0.009 (0.032)	0.061 (0.077)	0.123 (0.157)	0.024 (0.068)	−0.066 (0.155)	0.002 (0.060)	0.210 (0.162)
Running water	0.026 (0.044)	0.118 (0.119)	−0.024 (0.119)	0.069 (0.112)	−0.050 (0.101)	−0.039 (0.105)	0.130 (0.127)
Toilet	0.014 (0.038)	−0.012 (0.177)	0.003 (0.107)	−0.044 (0.103)	0.039 (0.090)	0.037 (0.081)	−0.033 (0.091)
Sanitary conditions	0.092 ** (0.044)	0.191 (0.135)	0.184 (0.119)	−0.036 (0.133)	0.156 (0.102)	0.104 (0.102)	0.021 (0.114)
SER	0.719	0.672	0.726	0.746	0.745	0.699	0.770
R^2^	0.011	0.063	0.010	0.012	0.033	0.058	−0.133

Note: *, **, and *** indicate significance at the 10%, 5%, and 1% levels, respectively; the values in parentheses are robust standard errors.

**Table A9:** Results of two-stage least squares (by age and residence, for physical health).

	Full Sample N = 3335	Urban Youth N = 577	Rural Youth N = 445	Urban Middle Age N = 610	Rural Middle Age N = 509	Urban Elderly N = 755	Rural Elderly N = 439
Intercept Term	3.167 *** (0.181)	3.505 *** (0.542)	3.653 *** (0.481)	2.831 *** (0.566)	2.161 *** (0.540)	3.011 *** (0.579)	3.340 *** (0.475)
Years of education	0.047 *** (0.011)	0.035 (0.045)	0.071 * (0.041)	0.018 (0.048)	0.176 * (0.091)	0.058 ** (0.025)	0.161 ** (0.073)
Sex	−0.069 ** (0.035)	−0.002 (0.073)	−0.099 (0.108)	−0.040 (0.085)	−0.318 (0.277)	−0.190 ** (0.076)	−0.338 (0.217)
Family income	0.028 (0.018)	0.006 (0.056)	−0.000 (0.060)	0.051 (0.050)	0.060 (0.047)	0.038 (0.046)	−0.005 (0.041)
Medical insurance	−0.171 ** (0.084)	0.244 (0.224)	−0.117 (0.199)	−0.451 ** (0.203)	−0.552 * (0.295)	−0.221 (0.207)	0.006 (0.290)
Medical institution	−0.335 *** (0.062)	−0.368 (0.271)	−1.177 (0.771)	−0.225 * (0.133)	−0.410 (0.252)	−0.431 *** (0.088)	0.170 (0.263)
Marital status	−0.003 (0.040)	−0.091 (0.109)	−0.083 (0.123)	0.253 ** (0.126)	0.289 (0.242)	0.041 (0.087)	−0.409 ** (0.186)
Job	0.015 (0.037)	−0.026 (0.225)	0.057 (0.124)	−0.035 (0.093)	−0.196 (0.143)	0.215 ** (0.099)	0.020 (0.091)
Working hours	0.024 * (0.012)	0.009 (0.031)	0.041 (0.037)	0.029 (0.030)	0.013 (0.045)	0.028 (0.025)	−0.004 (0.040)
Individual income	0.001 (0.012)	−0.040 (0.049)	−0.030 (0.029)	0.047 (0.074)	0.019 (0.024)	−0.021 (0.063)	−0.024 (0.030)
Smoking	0.045 (0.037)	−0.004 (0.113)	0.187 (0.119)	0.025 (0.108)	0.011 (0.121)	0.108 (0.077)	−0.057 (0.106)
Drinking	0.041 (0.034)	0.108 (0.091)	−0.049 (0.097)	−0.024 (0.079)	−0.076 (0.123)	0.184 *** (0.071)	0.013 (0.111)
Running water	0.112 *** (0.043)	−0.031 (0.180)	0.001 (0.094)	0.086 (0.171)	0.095 (0.105)	0.265 * (0.159)	0.382 *** (0.128)
Toilet	−0.098 ** (0.042)	0.044 (0.121)	−0.112 (0.113)	−0.115 (0.086)	−0.142 (0.149)	−0.117 (0.077)	−0.138 (0.116)
Sanitary conditions	−0.013 (0.045)	0.155 (0.196)	0.019 (0.098)	0.056 (0.164)	−0.145 (0.112)	0.002 (0.156)	−0.042 (0.115)
SER	0.712	0.686	0.732	0.714	0.942	0.711	0.851
R^2^	0.025	0.007	−0.085	0.043	−0.574	0.040	−0.484

Note: *, **, and *** indicate significance at the 10%, 5%, and 1% levels, respectively; the values in parentheses are robust standard errors.

**Table A10:** Results of two-stage least squares (by age and residence, for psychological health).

	Full Sample N = 3336	Urban Youth N = 581	Rural Youth N = 443	Urban Middle Age N = 609	Rural Middle Age N = 511	Urban Elderly N = 749	Rural Elderly N = 443
Intercept Term	2.572 *** (0.183)	3.981 *** (0.600)	2.176 *** (0.432)	2.671 *** (0.604)	2.616 *** (0.409)	2.933 *** (0.596)	1.994 *** (0.397)
Years of education	0.029 ** (0.011)	0.086 * (0.051)	−0.002 (0.038)	−0.000 (0.050)	0.042 (0.069)	0.061 ** (0.026)	−0.023 (0.059)
Sex	−0.102 *** (0.036)	−0.007 (0.081)	0.013 (0.096)	−0.111 (0.091)	−0.152 (0.211)	−0.229 *** (0.078)	−0.095 (0.175)
Family income	0.007 (0.018)	−0.139 ** (0.062)	0.013 (0.054)	0.016 (0.054)	0.023 (0.036)	0.037 (0.047)	0.096 *** (0.035)
Medical insurance	0.294 *** (0.085)	0.141 (0.247)	0.324 * (0.180)	0.712 *** (0.217)	−0.138 (0.225)	0.489 ** (0.213)	0.149 (0.241)
Medical institution	0.006 (0.063)	0.440 (0.300)	−0.109 (0.685)	−0.046 (0.144)	−0.140 (0.192)	−0.096 (0.091)	0.306 (0.213)
Marital status	0.058 (0.041)	0.099 (0.122)	0.114 (0.108)	0.028 (0.136)	−0.069 (0.181)	0.014 (0.090)	0.216 (0.151)
Job	−0.043 (0.037)	0.102 (0.249)	0.178 (0.111)	0.040 (0.097)	−0.010 (0.108)	0.055 (0.101)	0.041 (0.076)
Working hours	0.006 (0.012)	−0.012 (0.034)	−0.043 (0.033)	0.035 (0.032)	−0.010 (0.034)	0.011 (0.026)	0.021 (0.033)
Individual income	0.016 (0.012)	−0.023 (0.054)	0.036 (0.025)	−0.007 (0.078)	0.041 ** (0.018)	−0.076 (0.065)	0.005 (0.025)
Smoking	0.042 (0.037)	0.012 (0.125)	−0.094 (0.107)	0.050 (0.116)	0.024 (0.092)	0.121 (0.079)	0.150 * (0.089)
Drinking	0.033 (0.034)	0.049 (0.100)	0.056 (0.086)	−0.012 (0.085)	0.028 (0.094)	0.062 (0.073)	−0.011 (0.092)
Running water	0.014 (0.043)	−0.251 (0.200)	0.108 (0.083)	0.074 (0.182)	−0.008 (0.080)	0.015 (0.164)	−0.045 (0.107)
Toilet	−0.005 (0.043)	−0.145 (0.138)	0.136 (0.102)	−0.029 (0.091)	−0.022 (0.114)	0.034 (0.080)	0.016 (0.097)
Sanitary conditions	0.089 ** (0.045)	0.143 (0.217)	0.177 ** (0.087)	−0.050 (0.175)	0.101 (0.085)	−0.099 (0.160)	0.167 (0.096)
SER	0.716	0.760	0.649	0.761	0.718	0.730	0.707
R^2^	0.020	−0.071	0.094	0.024	0.032	−0.062	0.036

Note: *, **, and *** indicate significance at the 10%, 5%, and 1% levels, respectively; the values in parentheses are robust standard errors.
